# Adolescents with Intellectual Disability and Suicidal Behavior

**DOI:** 10.1100/tsw.2005.90

**Published:** 2005-09-08

**Authors:** Joav Merrick, Efrat Merrick, Mohammed Morad, Isack Kandel

**Affiliations:** ^1^ National Institute of Child Health and Human Development, Ben Gurion University of the Negev, Beer-Sheva, Israel; ^2^ Division of Pediatrics, Faculty of Health sciences, Ben Gurion University of the Negev, Beer-Sheva, Israel; ^3^ Center for Multidisciplinary Research in Aging, Faculty of Health sciences, Ben Gurion University of the Negev, Beer-Sheva, Israel; ^4^ Office of the Medical Director, Division for Mental Retardation, Ministry of Social Affairs, Jerusalem, Israel; ^5^ Clalit Health Services, Faculty of Health Sciences, Ben Gurion University of the Negev, Beer-Sheva, Israel; ^6^ Division for Community Health, Faculty of Health Sciences, Ben Gurion University of the Negev, Beer-Sheva, Israel; ^7^ Faculty of Social Science, Department of Behavioral Sciences, Academic College of Judea and Samaria, Ariel, Israel

**Keywords:** Adolescence, suicide, intellectual disability, developmental disability, mental retardation, human development, holistic health, public health, Israel

## Abstract

It has been assumed that impaired intellectual capacity could act as a buffer to suicidality in the population of children and adolescents with intellectual disability. The few studies that have been conducted contest this assumption, and in fact, the findings showed that the characteristics of suicidality in the population of children and adolescents with intellectual disability are very similar to other adolescents without intellectual disability. This paper reviews the few studies conducted and describe the symptomatology in this population.

## INTRODUCTION

Both children, adolescents and adults with intellectual disability (ID) and developmental disabilities (DD) are at high risk for developing mental health problems with a prevalence of psychopathology approximately four times higher than that found in the general population[1-3], but there is still a tendency to under diagnose psychiatric disorders in this population. This is due to diagnostic overshadowing, lack of appropriate diagnostic criteria and appropriate assessment measures. People with ID also have a higher incidence of depression[3,4], and it is therefore interesting that the issue of suicide in this population has received very little interest by researchers[5]. The prevalence of suicide and suicide attempts in this population seems much lower, but it does occur[4,5].

## SUICIDAL BEHAVIOR IN CHILDREN AND ADOLESCENTS WITH INTELLECTUAL DISABILITY

If there is very little research on suicide in adults with intellectual disability (ID), then there is only a handful of papers concerning suicide in children and adolescents with ID, which are for the most part case reports.

Sternlicht et al[6] reviewed the charts for all residents of a state school for persons with ID and found 12 adolescents (mean IQ 63, range 48-79) who had attempted suicide, or revealed suicidal ideation. Kaminer et al [7] reported on three adolescents with mild/moderate ID with suicidal ideation, including a 16 year old with mild ID and schizoaffective disorder who threatened to kill himself, since he was seeing and hearing scary things.

They also speculated that maybe intellectual disability and its intellectual and adaptive limitations could work as a “buffer” against suicidal behavior, because of the lack of cognitive sophistication to conceptualize, plan or carry out suicide. If this holds true, then this could be the reason for the lack of papers on this topic with this population, including children, adolescents and adults.

Menolascino et al[8] described eight persons with suicidal behavior out of 305 persons referred to an inpatient psychiatric facility. One 19 year old with mild ID was referred after his parents found him in his apartment with a knife and a suicide note.

A 35 year follow-up study on suicide mortality in a Finnish national cohort of 2,369 persons with ID[9] found ten cases of suicide and eight cases of undetermined external causes (UEC) of death, but none were below the age of 25 years. Apart from the above case reports, we have only found two other studies which we will describe in more detail.

## RHODE ISLAND STUDY

Walters et al[10], from the Department of Psychiatry and Human Behavior at the Brown University School of Medicine in Rhode Island, studied 90 consecutive admissions to their dual diagnosis specialty unit at the children’s psychiatric hospital. Nineteen adolescents (10 males) with a mean age of 15.75 years and a mean IQ of 59 (range 37-86) were identified as suicidal (21% of the sample). As part of the extensive assessment, coding and observation during the hospitalizations (mean length 11.3 months), descriptions of suicidality were classified as:

- Ideation as verbal statements about death, dying or killing oneself, but without expressed intent to do so (like: “I do not want to be in this world anymore. I want to be dead.”).
- Threat as verbal statements about intent to hurt or kill oneself with no associated behavior (like: “I am going to choke myself until I die.”).
- Behavior as potentially harmful actions with/without verbal statements (like running from the unit towards a busy street after saying that he/she want to be dead).

A large proportion (79%) of these 19 adolescents had prior psychiatric hospitalizations (mean of 2.4), and their psychopathology could be classified into three categories: behavioral disorders (six cases), affective disorders (five cases), psychotic disorders (one case) and family conflicts (seven cases). In fact, 10% of the adolescents had been physically abused, 10% sexually abused and 26% both physical and sexually abused. Thirty-two percent had a history of suicidality prior to or at the time of admission, 26% were suicidal only during hospitalization and 42% were suicidal both prior to and during hospitalization.

Out of the 19 adolescents, six were suicidal prior to admission and all expressed suicidal ideation, four made suicidal threats (66.6%) and three demonstrated suicidal behavior (50%). Of the other 13 who were suicidal only during hospitalization, or both prior to and during hospitalization, twelve (92.3%) expressed ideation, eleven (84.6%) made suicidal threats and nine (69.2%) demonstrated suicidal behavior.

This sample of 19 adolescents with mild-moderate intellectual disability showed suicidality characteristics similar to adolescents without intellectual disability, which, therefore, disputes the earlier assumptions[7] of intellectual disability serving as a “buffer” against suicidal behavior in this population. There was a high prevalence of physical or sexual abuse of the adolescent with intellectual disability prior to hospitalization, which could be the trigger of suicidal behavior also seen in the general population. Another earlier study[11] found that 39% of multihandicapped children admitted to a psychiatric hospital were reported to have been abused prior to the hospitalization.

## PITTSBURGH MEDICAL CENTER STUDY

Hardan and Sahl[12], from Western Psychiatric Institute and Clinic at University of Pittsburgh Medical Center, conducted a retrospective study of 233 patients over a 12 month period in their special program for children and adolescents with developmental and co-morbid psychiatric disorders (after school partial hospitalization program, summer intensive treatment program, school based partial program, a 24 bed inpatient unit and an out-patient clinic).

They found that 47 (20%) (34 males, 13 females, mean age 10, range 4-18 years) had a past or present history of suicide ideation or attempt (SI/SA). Of the 47, there were 12 (25.5%) with borderline, 17 (36%) with mild and 5 (11%) with moderate intellectual disability. There were 22 in the total sample of 233 with severe/profound intellectual disability, but none had SI/SA. The observation period for this study was 12 months.

Most of the 47 (94%) had had either SI only (13%), or in combination with threats and/or attempts, but only in four cases could it be said that the patient had an understanding of the concept of death and only one had a clear comprehension. Their psychopathology was mostly behavioral disorders (28 cases), affective disorders (16 cases), psychotic disorders (one case) and family conflicts (17 cases). In this study, there is no mention of past or present physical or sexual abuse, but that does not mean that it did not take place.

Eight adolescents (17%) had suicidal ideation on admission, or experienced ideation during hospitalization, and in 23 cases (49%) an acute psychosocial stressor was associated with SI/SA (there is no mention of what this psychosocial stressor was, but it could very well be abuse as was found in other studies[10,11]).

The most observed behaviors in the suicidal group were impulsivity, poor concentration, hyperactivity, sadness, aggression and sleep disturbances. The three most often observed symptoms were sadness, somatization and eating disturbances.

## EXPERIENCE FROM ISRAEL

The Division for Mental Retardation (DMR) of the Ministry of Social Affairs provides service to about 25,000 persons with intellectual disability in Israel[13]. About 6,500 are provided service in residential care centers, about 2,000 in community living (hostels, protected apartments), while the rest are provided service, but live with their families[13].

The Office of the Medical Director has every case of death in residential, or community care, reported in order to review all cases and decide whether further investigation is warranted[14]. For the 1991-2005 period there have been no cases of suicide in this population[14,15].

## DISCUSSION

Suicide and suicide attempts in children and adolescents with intellectual disability is a topic that has barely been studied by professionals working with this population, and therefore, has been thought to be a rare phenomenon. The two studies mentioned above[10,12], from two psychiatric settings in the United States catering to the population of children/adolescents with developmental and intellectual disability, from the 1995-1999 period, showed a frequency of 20-21% of suicidal behavior. These studies also showed that this behavior was more frequent in the inpatient setting, as a consequence of the worst cases being hospitalized.

In both studies[10,12], the characteristics, sex distribution and methods of suicide ideation and attempts were similar to that of adolescents without intellectual disability, but both studies had no case of completed suicide. The first study[10] had a high number of adolescents who were abused prior to admission, while the second study did not report abuse. This information (as in the general adolescent population) should alert every professional to investigate every case of attempted suicide in an adolescent for possible prior abuse (physical or sexual).

We would like to see further research of suicidal behavior in the population of adolescents with intellectual disability in order to learn more about the reasons for the very few cases of suicide reported and to learn more about further prevention and intervention techniques.

## CONCLUSIONS

The clinical implication of the above studies[10,12] are that suicidal ideation and attempts do occur in the population of children and adolescents with intellectual disability. Professionals should, therefore, be aware of and assess for this behavior. Sadness, or depression, are symptoms that could indicate later suicidal behavior.

## BIOSKETCHES

**Joav Merrick, MD, DMSc,** is professor of child health and human development affiliated with the Center for Multidisciplinary Research in Aging, Zusman Child Development Center, Division of Pediatrics and Community Health at the Ben Gurion University, Beer-Sheva, Israel, the medical director of the Division for Mental Retardation, Ministry of Social Affairs, Jerusalem, the founder and director of the National Institute of Child Health and Human Development. Has written numerous publications in the field of child health and human development, rehabilitation, intellectual disability, disability, health, welfare, abuse, advocacy, quality of life and prevention. Received the Peter Sabroe Child Award for outstanding work on behalf of Danish Children in 1985 and the International LEGO-Prize ("The Children's Nobel Prize") for an extraordinary contribution towards improvement in child welfare and well-being in 1987. E-Mail: jmerrick@internet-zahav.net. Website: www.nichd-israel.com

**Efrat Merrick,** is a medical student at the Sackler School of Medicine, Tel Aviv University and a research assistant at the National Institute of Child Health and Human Development, Faculty of Health Sciences, Ben Gurion University of the Negev, Beer-Sheva, Israel. E-mail: efratmerrick@gmail.com

**Mohammed Morad,** MD, is specialist in family medicine, lecturer in family medicine affiliated with the National Institute of Child Health and Human Development, Division of Community Health and Center for Multidisciplinary Research in Aging, Faculty of Health Sciences, Ben Gurion University of the Negev and the medical director of a large area clinic in the city of Beer-Sheva operated by the Clalit Health Services. Publications on Bedouin health, health aspects, spiritual health and aging in persons with intellectual disability, and presenter on topics like health policy and services for disadvantaged at national and international conferences. E-mail: morad62@013.net.il

**Isack Kandel, MA, PhD,** is senior lecturer at the Faculty of Social Sciences, Department of Behavioral Sciences, the Academic College of Judea and Samaria, Ariel. During the period 1985-93, he served as the director of the Division for Mental Retardation, Ministry of Social Affairs, Jerusalem, Israel. E-mail: kandelii@zahav.net.il
